# Canonical Wnt Signaling Drives Tumor-Like Lesions from Sox2-Positive Precursors of the Murine Olfactory Epithelium

**DOI:** 10.1371/journal.pone.0166690

**Published:** 2016-11-30

**Authors:** Nils W. Engel, Julia E. Neumann, Julia Ahlfeld, Annika K. Wefers, Daniel J. Merk, Jasmin Ohli, Ulrich Schüller

**Affiliations:** 1 Center for Neuropathology, Ludwig-Maximilians-University, Munich, Germany; 2 Department of Internal Medicine II, University Medical Center, Hamburg-Eppendorf, Germany; 3 Institute of Neuropathology, University Medical Center, Hamburg-Eppendorf, Germany; 4 Research Institute Children’s Cancer Center, Hamburg, Germany; 5 Department of Pediatric Hematology and Oncology, University Medical Center, Hamburg-Eppendorf, Germany; University of Kentucky, UNITED STATES

## Abstract

Canonical Wnt signaling is known to promote proliferation of olfactory stem cells. In order to investigate the effects of a constitutive activation of Wnt signaling in Sox2-positive precursor cells of the olfactory epithelium, we used transgenic mice that allowed an inducible deletion of exon 3 of the *Ctnnb1* gene, which is responsible for the phosphorylation and degradation of Ctnnb1 protein. After induction of aberrant Wnt activation by Ctnnb1 deletion at embryonic day 14, such mice developed tumor-like lesions in upper parts of the nasal cavity. We still observed areas of epithelial hyperplasia within the olfactory epithelium following early postnatal Wnt activation, but the olfactory epithelial architecture remained unaffected in most parts when Wnt was activated at postnatal day 21 or later. In summary, our results suggest an age-dependent tumorigenic potential of aberrant Wnt signaling in the olfactory epithelium of mice.

## Introduction

The mammalian olfactory epithelium (OE) is a sensory neuroepithelium, which is unique for its ability of sustaining robust peripheral neurogenesis throughout the lifetime of mice and man [1–4]. The complex regulatory pathways underlying this physiologic capacity have been a matter of intensive biomedical research, given the hope, that major insights here might sustainably transform the field of regenerative medicine in the future. Only recently, the crucial role of the canonical Wnt signaling pathway in the regulation of OE stem cells and progenitors during development and regeneration has been pointed out [5, 6]. Wang et al. showed, that Wnt signaling promotes self-renewal of Sox2-positive stem cells and proximate progenitors of the OE *in vitro* [5]. Moreover, Wnt signaling was found to promote neurogenesis at the expense of non-neuronal differentiation within the OE. Following OE injury in adult mice, strong Wnt activation seems to be necessary for OE regeneration *in vivo*. In this context, Chen et al. confirmed that Wnt activation may have a pivotal role in OE progenitor cell proliferation and neurogenesis *in vivo*, and they further suggested that Wnt signaling delays the terminal differentiation of neuronal progenitors into mature olfactory sensory neurons, therefore promoting the expansion of OE immature neuronal progenitors [6].

Beyond the physiological role of Wnt signaling for the regulation of cellular growth and differentiation throughout the body, pathological activation of the canonical Wnt signaling pathway has been identified to play a crucial role in a wide range of human neoplastic diseases [7–13]. Of note, the Wnt pathway hereby often takes up important physiologic functions and has devastating tumor-causing potential in cases of dysregulation within the same tissue. This is exemplarily true for intestinal tissue homeostasis [14, 15] and colon cancer formation [16–18]. In the central nervous system, Wnt signaling has for instance crucial roles in hindbrain development [19–22] and may induce WNT medulloblastoma formation [12]. This relation is even generalizable to the well-established link between the essential role of certain signaling pathways for brain development and their contribution to the pathogenesis of various human pediatric brain tumors [23].

Here, we sought to answer the question, whether the above outlined parallelism is valid in an OE related context, which would imply a tumorigenic potential of aberrant Wnt signaling within the OE. Thus, we constructed an inducible *Sox2-creER*^*T2*^::*Ctnnb1(ex3)*^*Fl/+*^ mouse model in order to effectively delete exon 3 of *Ctnnb1* in Sox2-positive cells of the mouse OE. The resulting alterations in OE architecture were then analyzed on the level of histomorphology and immunohistochemistry.

## Material and methods

### Transgenic mice

*Sox2-creER*^*T2*^ [24] and *B6*.*Cg-Gt(ROSA)26Sor*^*tm9(CAG-tdTomato)Hze/J*^ [25] mice were obtained from the Jackson Laboratory. *Ctnnb1(ex3)*^*Fl/FL*^ [26] mice were a kind gift from Dr. M. Taketo (University of Tokyo, Japan). Genotyping was performed by PCR analysis using genomic DNA from ear biopsies. Primers for *Cre* and *Ctnnb1(ex3)* have been previously published [26, 27], and primers to detect the *tdTomato* allele have been designed as recommended by the Jackson Laboratory (www.jax.org). *In vivo* induction of the *tdTomato* or the defective *Ctnnb1(ex3)* allele in Sox2-positive cells was achieved by intraperitoneal tamoxifen (Sigma-Aldrich) injection. Pregnant females were injected once with 1 mg of tamoxifen (50 mg/kg body weight in corn oil (Sigma-Aldrich) at E14.5. The day of vaginal plug detection was considered E0.5. Postnatal mice were induced at days P7, P14 or P21 via single tamoxifen injection, likewise. Injected mice were monitored daily for signs of tumor formation or general failure to thrive. Together with littermates not bearing the *Cre* allele, mutant animals destined for further histological workup were sacrificed shortly after the onset of symptoms by cervical dislocation. All mouse procedures were performed according to protocols approved by the government of Upper Bavaria (#2532-10-14). All efforts were made to ameliorate suffering of mice. Mice were sacrificed by cervical dislocation as soon as any kind of suffering, deterioration of the general health condition or neurological symptoms were visible. This given, analgesics or anesthetics were not applied in order to reduce suffering. Mice were monitored at least once a day. There were no unexpected deaths within this study.

### Tissue collection, fixation and H&E staining

Sacrificed animals destined for histological evaluation were immediately dissected for tissue collection. The skull bases were formalin-fixed, decalcified in 10% EDTA (pH7.4) and embedded in paraffin according to standard lab protocols. For each of those animals, brain, heart, lungs, gastrointestinal organs and kidneys were collected and separately fixed and embedded according to standard lab protocols. The preserved skull base specimens were cut in frontal orientation until a level with intersection of ethmoturbinates 1–5 was reached. Sections from this level were collected and H&E stains were acquired according to standard lab protocols. For all other organs, representative H&E stains in suitable orientation were evaluated for histomorphological alterations in mutant organs compared to controls. If alterations were detectable, further immunohistochemical staining was performed as described below.

### Immunohistochemistry

Sections of paraffin-embedded tissue were deparaffinized and rehydrated before heat-induced antigen retrieval was conducted at 100°C for 20 min in 10 mM sodium citrate buffer for all antibodies. Immunohistochemical staining was done using the primary antibodies in concentrations as follows: RFP: 1:200, Antibodies Online, Cat N° AA234; Sox2: 1:200, Abcam, Cat N° AB79351; Ki67: 1:200, Abcam, Cat N° AB16667, Beta-Catenin: 1:1000, BD Pharmigen, Cat N° 610153; Mash1: 1:25, BD Bioscience, Cat N° 556604; Chromogranin A: 1:300, Abcam, Cat N° AB15160; CD56: undiluted, Ventana Medical Systems, Roche, Cat N° 7602625; S100: 1:2000, DAKO, Cat N° Z0311; SMA: 1:1000, DAKO, Cat N° M0851. Mash1 antibody was applied in blocking puffer over night at room temperature [28]. All other antibodies were applied over night at 4°C. The HRP/DAB Staining System (DAKO) was used according to the manufacturer’s specifications. Hemalaun was used for nuclear counterstaining. All histological photomicrographs were taken digitally using an Olympus BX50 microscope in combination with the Color View Soft imaging system. For immunofluorescent staining, sections were washed twice with PBS/0.1% Triton X-100 and then incubated in blocking buffer (I-Block protein- based blocking reagent; Applied Biosystems) for 30 minutes. Primary antibodies (RFP: 1:200, Antibodies Online, Cat N° AA234; Sox2: 1:200, Abcam, Cat N° AB79351; Tuj1: 1:100, BABCO, Cat N° MMS435P) were diluted in blocking buffer and applied over night at 4°C. Next, sections were washed twice with PBS/0.1% Triton X-100 and incubated for another 60 minutes with a 1:500 dilution of fluorescence-labeled secondary antibodies (goat anti-rabbit Alexa546; goat anti-mouse Alexa488, Invitrogen) in blocking buffer. Sections were washed twice with PBS/0.1% Triton X-100, counterstained with 4’,6-diamidino-2-phenylindole (DAPI), and mounted in Fluorescent Mounting Medium (DAKO). Images of tissue sections were collected on a Zeiss LSM 780 laser scanning microscope in combination with the ZEN 2012 imaging software. Whole-mount images of stomachs were collected on a Leica DFC3000 G microscope camera in combination with the Leica Application Suite Software.

### Statistical analysis

Data of survival, body weight and body seize of mice were analyzed using the Prism5 software (GraphPad). Survival of mice was analyzed using Kaplan–Meier survival curves, and the log rank test was used to examine the significance of results. P-values <0.05 were considered significant. For the analysis of body size and body weight of mice, the unpaired t-test was applied to compare the means of two groups with assumed Gaussian distribution and equal variances. All respective histograms illustrate the standard error of the mean (SEM; error bars).

## Results

### The main cell types of the mature OE originate mainly from Sox2-positive progenitor cells

**Fig 1:**
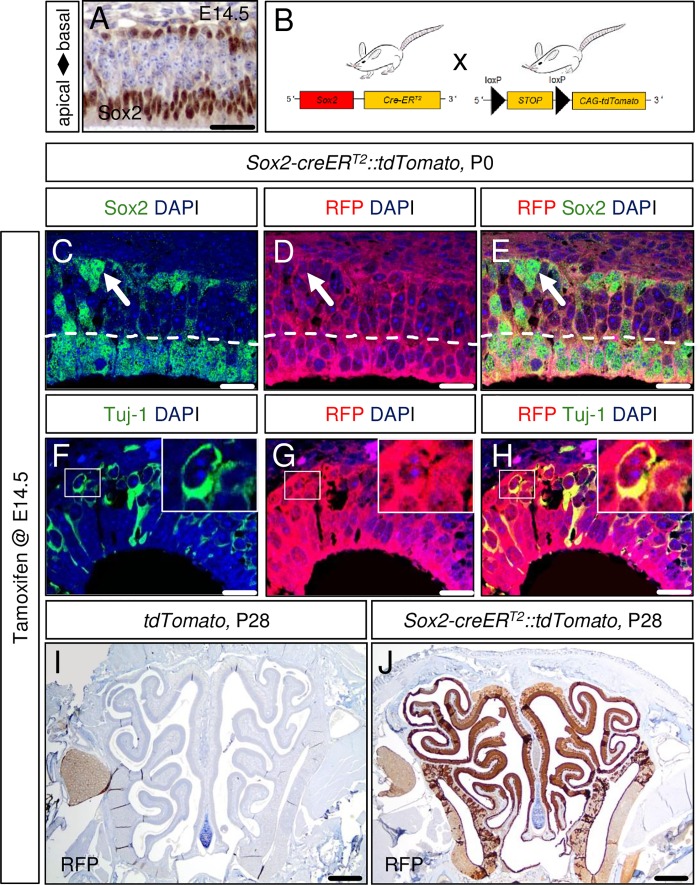
The *Sox2-CreER*^*T2*^ mouse is a suitable driver strain for studying gene function in the mouse olfactory epithelium (OE). At embryonic day 14.5 of mouse development (E14.5) the transcription factor Sox2 is already expressed in basal and apical progenitor cells of the olfactory epithelium (A). The mouse breeding scheme used in this study is displayed in panel B. Model induction via single tamoxifen application at E14.5 targets the whole progeny of the OE structure present at the day of birth (P0) as demonstrated in a *Sox2-creER*^*T2*^::*tdTomato* reporter strain (D). These targeted cells comprise Sox2-positive stem cells at the basal site of the OE (arrows, C-E), non-neuronal apical Sox2-positive sustentacular cells (nuclei of sustentacular cells delineated by dashed line, C-E) as well as Tuj-1-positive neuronal cells of the OE (a Tuj1/RFP-double-positive cell is depicted in high power insets, F-H). Single embryonic tamoxifen injection of *Sox2-creER*^*T2*^::*tdTomato* animals is sufficient to cover the whole mature OE in adult mice as shown for P28 (J; specific RFP detection compared to control in I). Scale bars equate to 500 μm in I-J, 25 μm in A and 10 μm in C-H.

The mature OE structure is composed of neuronal and non-neuronal OE cells. As we did not know, which OE cell lineage might be susceptible for aberrant Wnt signaling, we aimed for a Cre-driver strain that broadly targets the OE. It is known that OE stem cell populations as well as OE sustentacular cells express the transcription factor Sox2 [29]. To investigate the suitability of a Sox2-driver for our purpose, we first examined the Sox2-expression within the OE of *wild type* mouse embryos. We regarded the mouse embryonic day E14.5 as an ideal time for this investigation and for the later Cre-driver-based fate-mapping studies within the OE, first, because the basic composition of the mature OE is known to be established between E13.5 and E15.5 [30] and, second, we expected the maximum impact of inserted mutations within the OE to be realized by early time point mutational induction. As expected, E14.5 mouse OE tissue shows remarkable Sox2 expression (Fig 1A) in mitotically active apical and basal progenitor cells, which include a putative stem cell pool [31].

Next, we wanted to examine how the progeny of these Sox2-positive cells contribute to the cellular composition of the olfactory epithelium. Therefore, we bred *Sox2-creER*^*T2*^ mice with a red fluorescent protein (RFP) variant (*tdTomato)* reporter strain (Fig 1B), induced this fate-mapping system via a single intraperitoneal tamoxifen injection of pregnant females at E14.5, and sacrificed the mice at the day of birth (P0). While immunohistochemical staining for RFP on OE tissue of Cre-negative animals was negative (data not shown), “effective” mutant mice revealed a homogenous staining of the whole OE structure, indicating that close to every cell within the OE at P0 stemmed from a Sox2-positive precursor cell (Fig 1D). These fate-mapped OE cells comprised all major cell types of the postnatal OE as demonstrated by double-immunofluorescent staining: Sox2/RFP-positive stem cells at the OE base (Fig 1C–1E, arrows), Sox2/RFP-positive sustentacular cells at the apical OE site (Fig 1C–1E, nuclei of sustentacular cells below dashed line) and Tuj-1/RFP-positive cells that belong to the neuronal OE lineage (Fig 1F–1H). Even in the mature OE at postnatal day P28, a homogenous and specific RFP staining throughout the OE was observed (Fig 1J; Fig 1I shows control), indicating that the stem cell pool, necessary for postnatal OE expansion and regeneration does not arise independently from Sox2-precursors at E14.5, but is a progeny of those. Interestingly, similar results are observed, if Tamoxifen is applied at P21, although the RFP staining at P42 is a little more heterogeneous in this situation (S1 Fig).

We concluded that no large cell population different from Sox2-positive progenitor cells, targetable as early as E14.5, contributes to the cellular composition of the mature OE. Therefore, the *Sox2-creER*^*T2*^ mouse appears to be a suitable driver strain for performing gene function studies within the OE, irrespective of cell type of interest. Likewise, E14.5 seemed to be an appropriate time point of a genetic manipulation in order to elicit a well-marked phenotype at postnatal days. As the late-embryonically established pattern of Sox2-expression within the OE remains preserved at postnatal stages of development [29], the postnatal OE structure even continues to be targetable for our model approach. Thus, we also considered model induction at postnatal days of development.

### Constitutive activation of the canonical Wnt signaling pathway at E14.5 leads to the formation of tumor-like lesions within the OE of mice

**Fig 2:**
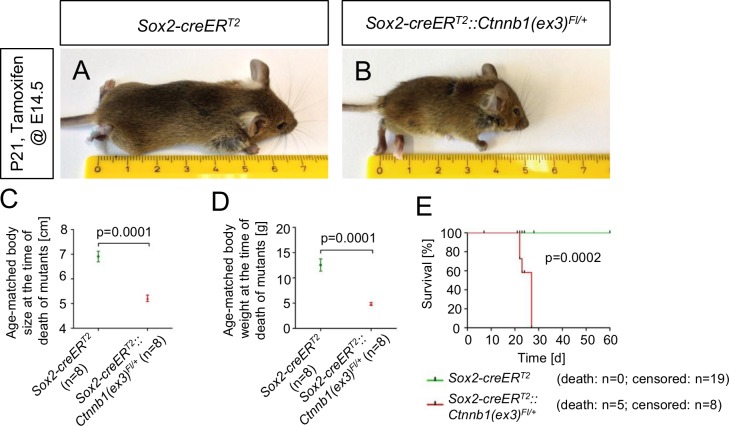
Embryonically induced mutant mice present with a failure to thrive and die prematurely when compared to controls. At E14.5 tamoxifen-induced *Sox2-creER*^*T2*^::*Ctnnb1(ex3)*^*Fl/+*^ mice develop a marked failure to thrive compared to controls (B vs. A) and show significant deficits in body size (C) and body weight (D) at their time of death. Mutant mice had to be sacrificed prematurely between postnatal days 21–28 (E).

To model a constitutive activation of the canonical Wnt signaling pathway within the OE, as being found in a plethora of human neoplastic diseases, we next bred *Sox2-creER*^*T2*^ mice to *Ctnnb1(ex3)*^*Fl/Fl*^ mice and induced the model via single intraperitoneal tamoxifen injection of pregnant females at E14.5. The offspring expressed an exon 3 depleted version of the Beta-Catenin protein, preventing its degradation [26] and resulting in a constitutive activation of the Wnt signaling pathway in Sox2-expressing cells at E14.5 and their progeny throughout the body. These *Sox2-creER*^*T2*^::*Ctnnb1(ex3)*^*Fl/+*^ mice developed a marked failure to thrive until the age of postnatal day P21 with significant deficits in body size and body weight compared to controls (Fig 2A–2D) and presented with general weakness and ataxia. Finally, all of these mice had to be sacrificed 3 to 4 weeks after birth (Fig 2E). An orienting histopathological review of the major organ systems of embryonically tamoxifen-injected mice, covering brain, heart, lungs, kidneys and the gastrointestinal tract revealed unusual small stomachs in mutants, bearing hyperplastic, lumen-narrowing lesions in glandular and non-glandular parts of the organ (S2 Fig; organ systems of postnatally induced mice not examined). These lesions might contribute to malnutrition and malabsorption, which would offer a plausible explanation for the phenotype of mutant mice.

**Fig 3:**
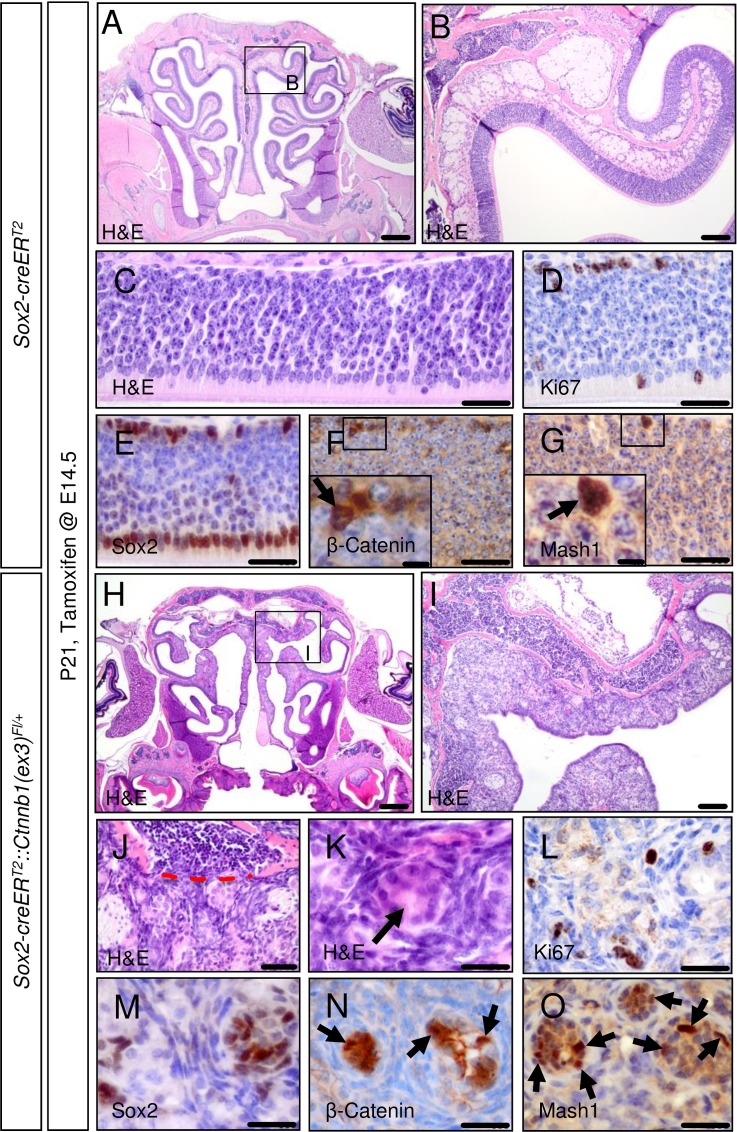
Embryonically activated aberrant Wnt signaling in *Sox2-creER*^*T2*^::*Ctnnb1(ex3)*^*Fl/+*^ mice leads to the formation of tumor-like lesions within the olfactory epithelium. Tamoxifen application in *Sox2-creER*^*T2*^::*Ctnnb1(ex3)*^*Fl/+*^ mice at E14.5 causes aberrant activation of the canonical Wnt signaling pathway in Sox2-positive cells of the mouse OE and leads to the formation of tumor-like lesions within this structure. As shown for P21 mice by H&E staining, the structure of the native OE in mutant mice compared to controls is fully disrupted. Alterations are more pronounced in the upper nasal cavity (A, OE of control mice; B, higher magnification of framed area in A; H, OE tumor-like lesions of mutant mice; I, higher magnification of framed area in C). OE tumor-like lesions of mutant mice display signs of infiltration with disruption of bone laminae (C, control OE; J, red dashed line indicates broken bone barrier in mutant OE lesions) as a feature of malignant growth. Rosette-like structures are present in OE lesions of mutants (C, control OE; K, rosette in mutant OE lesion indicated by arrow). The amount of Ki67-positive cells in mouse OE tumor lesions remains comparable to the native OE (D, control OE; L, mutant OE lesion). Staining for Sox2, ß-Catenin and Mash1 delineate tumor cell nests from a surrounding stromal cell compartment (E, F, G, control stains; M, N, O, stains in mutant OE lesions). Scale bars equate to 500 μm in A and H, equate to 100 μm in B and I, equate to 25 μm in C-G and J-O and equate to 5 μm in insets.

**Fig 4:**
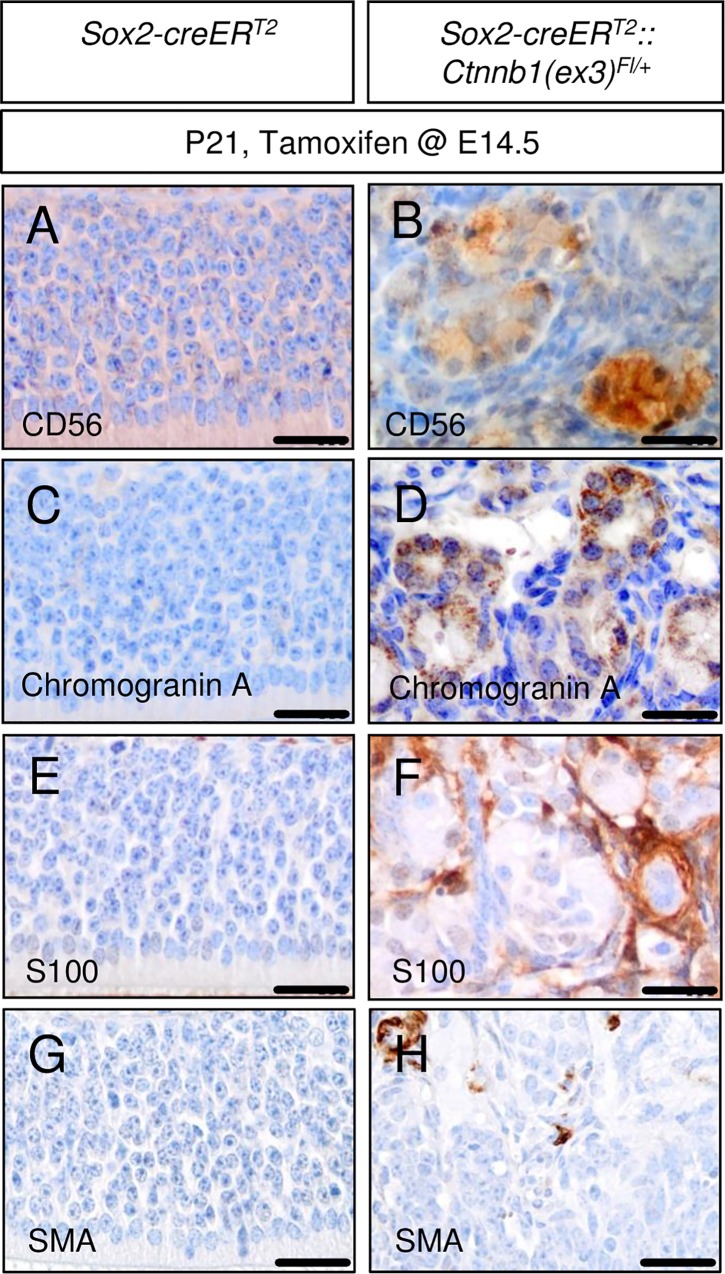
Additional immunohistochemical staining of tumor-like lesions in embryonically induced mutant mice suggest similarity to human olfactory neuroblastoma (hONB), but distinctness from human sinonasal haemangiopericytoma (sHPC). The expression of markers of neuroendocrine differentiation as CD56 and Chromogranin A and positivity for S100 are crucial requirements for the proper diagnosis of hONB and can be detected in OE mouse tumor-like lesions (B, D, F), but are found to be absent in the native mouse OE (A, C, E). SMA is known to be widely diffuse positive in sinonasal haemangiopericytoma but does not homogenously stain cells in our mouse tumor-like lesions (H) or native mouse OE (G). Scale bars equate to 25 μm.

The OE architecture of these mutant mice was demolished and had become replaced by tumorous tissue, slightly pronounced in the upper part of the nasal cavity (Fig 3H and 3I; Fig 3A and 3B shows control). These mouse OE lesions displayed signs of infiltration with disruption of bone laminae (Fig 3J) and rosette-like differentiation features (Fig 3K) compared to the normal mouse OE (Fig 3C). However, the amount of Ki-67-positive cells in the OE mouse tumor-like lesions appeared to be not visually altered compared to native OE tissue (Fig 3L vs. 3D). Immunohistochemical staining allowed the detection of Sox2, Mash1, a marker of early neuronal lineage restriction in the OE, and nuclear accumulation of Beta-Catenin, a correlate of activated canonical Wnt signaling, in the native P21 mouse OE (Fig 3E–3G). Mouse OE tumor-like lesions expressed these markers as well, but marker expression here seemed to delineate nests of stained cells surrounded by an unstained stromal cell compartment (Fig 3M–3O). Additional stains revealed expression of CD56, Chromogranin A, S100, but not Smooth muscle actin (SMA, Fig 4). In summary, the embryonical induction of aberrant Wnt signaling in the OE of mice lead to the formation of tumor-like lesions with a hinted nest-stroma cytoarchitecture.

### Postnatally induced aberrant Wnt signaling within the OE leads to an age-dependent formation of OE hyperplasia

**Fig 5:**
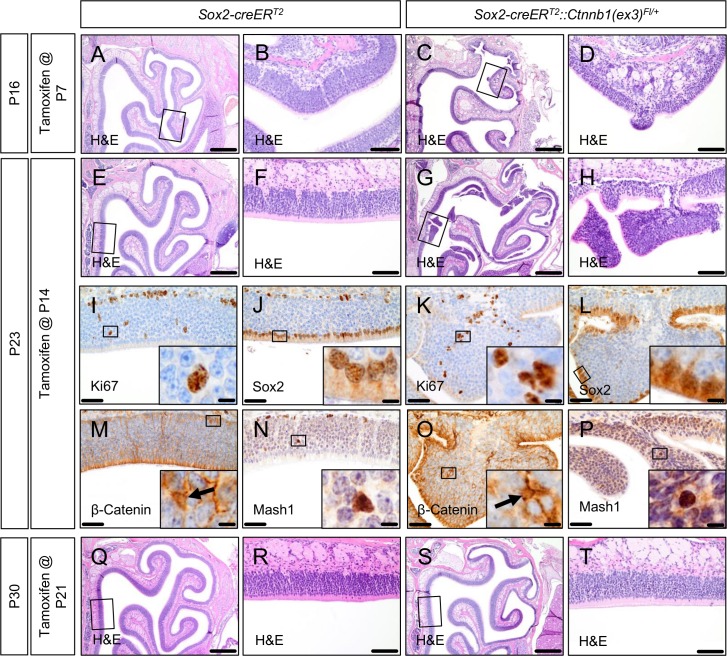
Early postnatally activated aberrant Wnt signaling in *Sox2-creER*^*T2*^::*Ctnnb1(ex3)*^*Fl/+*^ mice leads to the formation of epithelial hyperplasia within the OE. Tamoxifen application in *Sox2-creER*^*T2*^::*Ctnnb1(ex3)*^*Fl/+*^ mice at postnatal day 7 (P7) or 14 (P14) leads to the formation of OE hyperplasia (A, E, OE of control mice; B, F, higher magnification of framed areas in A, E showing normal OE; C, hyperplasia in P7-induced mutant mice; D, higher magnification of framed area in C; G, multiple areas of hyperplasia in P14-induced mutant mice captured on different slice levels; H, higher magnification of framed area in G). IHC staining patterns for Ki67, Sox2, ß-Catenin and Mash1 of exemplary areas of hyperplasia in P14-induced mutant mice are comparable to control OE, indicating a grossly normal cell differentiation and preserved OE polarity in such areas (I, J, M, N, IHC stains of control mice OE; K, L, O, P, respective IHC stains of hyperplasia; insets display high power magnifications of framed areas in I-P and exemplarily highlight positive cells for the respective marker). At postnatal day 21 (P21) tamoxifen-induced mutant mice do not develop those alterations anymore (Q-T). Scale bars equate to 500 μm in A, C, E, G, Q and S, equate to 100 μm in B, D, F, H, R and T, equate to 50 μm in I-P, and equate to 10 μm in insets.

In a next step we wanted to test, whether such OE tumor-like lesions would be reproducible by aberrant activation of the canonical Wnt signaling pathway in the OE of mice at postnatal stages of development. Therefore, we injected *Sox2-creER*^*T2*^::*Ctnnb1(ex3)*^*Fl/+*^ mice and *Sox2-creER*^*T2*^ mice as controls with single tamoxifen dosages at postnatal days P7, 14 or 21. Analogous to the embryonically injected mutant mice, postnatally induced mice developed a marked failure to thrive shortly after tamoxifen treatment and had to be sacrificed within 2 weeks after tamoxifen injection (data not shown). Compared with embryonically induced mutants, the OE cytoarchitecture of postnatally induced mice appeared to be grossly intact. However, P7 and P14 induced mutant mice presented with areas of epithelial hyperplasia arising from the otherwise normal OE structure (OE hyperplasia in a representative P7 induced mutant in Fig 5C and 5D compared to control in Fig 5A and 5B; OE hyperplasia in a representative P14 induced mutant in Fig 5G and 5H compared to control in Fig 5E and 5F). Immunohistochemical staining of such OE hyperplasia for Ki67, Sox2, Beta-Catenin and Mash1 did neither lead to the assumption of a remarkably impaired cell differentiation nor a visibly increased proliferation rate in such lesions compared to controls (Fig 5I–5P). The basal-apical polarity of the native OE was largely preserved.

When mutant mice were induced not until P21, the OE of such mutant mice remained indistinguishable from the native OE of controls (Fig 5Q–5T) and hyperplasia was not observed.

Altogether, this age-dependent capacity of aberrant Wnt activation, which induces hyperplastic growth in the postnatal OE as well as the more tumor-like phenotype of OE lesions seen in embryonically induced mutants, may suggest an age-dependent tumorigenic potential of pathological aberrant Wnt signaling within the OE of mice.

## Discussion

We report here a mouse model system, in which the artificially induced constitutive activation of the canonical Wnt signaling pathway at E14.5 drives the formation of tumor-like OE lesions from Sox2-positive precursors of the OE structure. Furthermore, Wnt activation at early postnatal stages of mouse development leads to an OE hyperplasia without obvious malignant features.

As such, the tumorigenic potential of pathological activated Wnt signaling within the murine OE seems to fade out with age and completely disappears at the age of about 3 weeks. These results are not unexpected, as massive OE cell proliferation is a physiological process in embryonic and early postnatal OE development [32], and one would expect a generally more permissive microenvironment for proliferation during these stages. Around 30 days after mouse birth, this mode of cell expansion finally slows down into a state of OE maintenance [32] with consecutively low cell-turnover, leading to the assumption of a then more proliferation-suppressing environment.

Interestingly, even the tumorous lesions observed after Wnt activation in the developing murine OE et E14.5 do not demonstrate a grossly enhanced proliferation rate compared to controls. However, this finding does not necessarily vote against the neoplastic character of the lesion. Olfactory neuroblastoma, for instance, that are believed to arise from the olfactory epithelium, do neither proliferate extensively [33]. Furthermore, canonical Wnt signaling is known to have the capability to block apoptosis, as demonstrated in mesenchymal cells [34, 35]. It therefore appears possible that aberrant Wnt signaling in the olfactory epithelium disturb the physiological cell turnover by a blockade of apoptosis rather than significantly to enhance the rate of proliferation.

Obvious similarities concerning neoplastic lesions of human head and neck pathology to our postnatally induced mouse OE hyperplasia, which reminded us of the classical adenoma-carcinoma sequence in colorectal carcinogenesis [36, 37], are missing. Sinonasal polyposis is a common disease in human beings, but is considered to produce benign hyperplastic lesions associated with chronic inflammation, rather than forming precursor lesions of future malignant transformation, driven by somatic or inherited mutations [38, 39]. Also, inflammatory nasal polyps are not likely to arise from the olfactory mucosa [40]. However, an upregulation of Wnt pathway genes in nasal polyposis has been reported [41].

A malignant human tumor that is thought to arise from the human OE [42] is the olfactory neuroblastoma (esthesioneuroblastoma), and we noticed some similarities of our embryonically induced OE tumor-like lesions and human olfactory neuroblastoma (hONB) on the level of histopathology, including positive staining for markers of neuroendocrine differentiation, such as CD56 and Chromogranin A (Fig 4A–4D). Similarly, S100 expression is used to differentiate hONB from neuroendocrine carcinoma [43, 44] and is detectable in our mouse OE lesions as well (Fig 4E and 4F). hONB is a rare sinonasal malignancy of neuroectodermal differentiation, but due to its rarity, very little is known about the molecular basis of ONB formation. However, nuclear accumulation of Beta-Catenin as a correlate of constitutive activation of the canonical Wnt signaling pathway, which is detectable in mouse OE tumor-like lesions and native mouse OE tissue, was not detectable in any of 27 analyzed *bona fide* hONB (unpublished observations).

In recent years, sinonasal haemangiopericytoma (sHPC) have been proposed as an independent tumor entity that is distinct from solitary fibrous tumors of the sinonasal tract and meningeal HPC [45]. Most notably, sHPC have been found to harbor frequent *CTNNB1* mutations [46, 47], ranking this tumor entity as a strong candidate for offering an equivalent to our mouse OE tumor-like lesions in human pathology. However, the histological phenotype of sHPC has been described to be quite different from what we encounter in our OE model lesions. In particular, one would expect a vascular endothelial differentiation in these probably pericyte-derived tumors instead of the rather neuroendocrine differentiation profile of our mouse OE lesions. Furthermore, strong and diffuse cytoplasmatic expression of smooth-muscle actin (SMA) is a consistent feature of human sHPC [45, 48], but is hardly detectable in our mutant mouse OE lesions (Fig 4G and 4H).

Owed to the limited lifespan of the introduced mouse model we described relatively small OE tumor-like lesions in this study, even after embryonic induction of Wnt-pathway activity. Nevertheless, the described lesions clearly indicate that a tight control on Wnt signaling is required in the developing olfactory epithelium. More work is required to understand, to which extent the variety of human sinonasal neoplasms may develop with the contribution of activated Wnt signaling and whether Sox2-positive precursors in the olfactory epithelium could indeed serve as a cellular origin for such tumors.

## Supporting Information

S1 Fig Immunostaining using antibodies against RFP on frontal sections from 42-day-old *Sox2-creERT2*::*tdTomato* mice with tamoxifen application at P21. Higher magnifications at the bottom display medial (left) and lateral (right) regions.
(TIF)

S2 Fig Embryonically induced mutant mice display small stomachs with hyperplastic lesions in glandular and non-glandular parts of the organ at the time of death. At E14.5 tamoxifen-induced *Sox2-creER*^*T2*^::*Ctnnb1(ex3)*^*Fl/+*^ mice display small stomachs at the time of death compared to controls (whole organ in C vs. A; sliced organ in D vs. B). The stomachs of mutant mice exhibit lumen-narrowing hyperplastic lesions in forestomach (G vs. E) and glandular stomach (H vs. F) parts. The epithelial cells of forestomach lesions show an endophytic growth pattern (K vs. I) with visually enhanced proliferation in Ki67 staining (O vs. M) in comparison to the native forestomach epithelium. The glandular lesions present altered cytoarchitecture and cell morphology compared to the native glandular stomach (L vs. J), whereas ß-Catenin staining intensity seems to be enhanced in these lesions (T vs. R; higher magnification of framed areas in insets). Ki67 staining in glandular lesions and native glands (P vs. N) and ß-Catenin staining in forestomach lesions and native forestomach (S vs. Q) is similar. Scale bars equate to 2 mm in A-D, equate to 500 μm in E-H, equate to 50 μm in I-P, equate to 20 μm in Q-T and equate to 10 μm in insets.
(TIF)
