# Supplementary figures and images for: Canonical Wnt Signaling Drives Tumor-Like Lesions from Sox2-Positive Precursors of the Murine Olfactory Epithelium
Source: PLoS One. 2016 Nov 30;11(11):e0166690. doi: 10.1371/journal.pone.0166690 (PMC5130221; doi:10.1371/journal.pone.0166690)

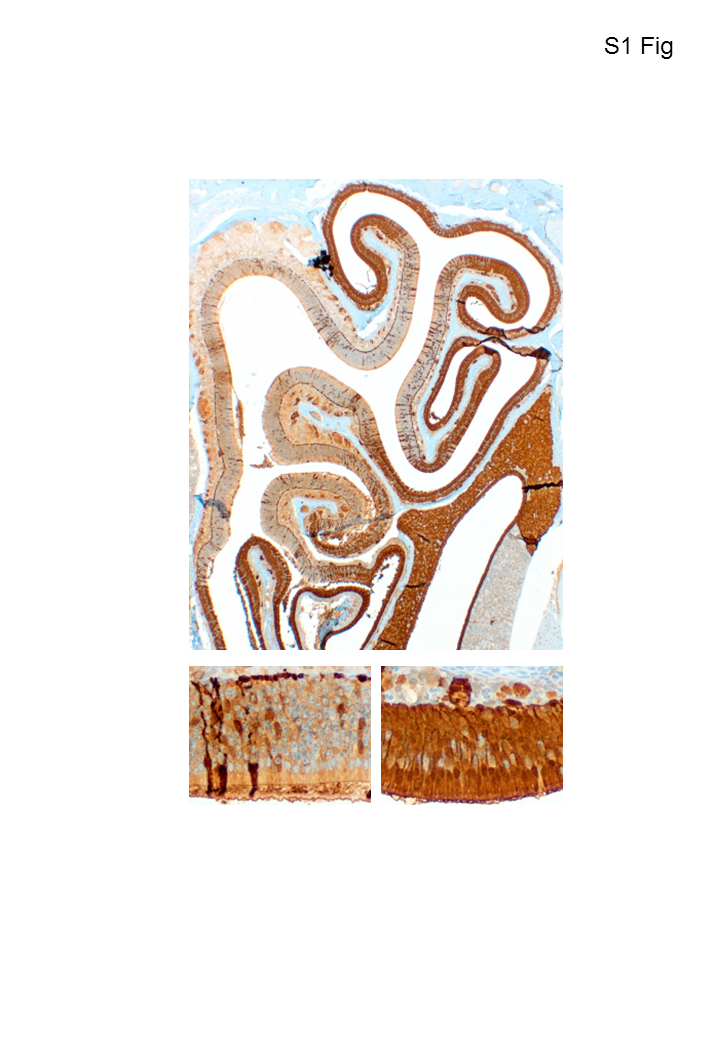

Supplement: S1 Fig — Higher magnifications at the bottom display medial (left) and lateral (right) regions. (TIF) [file pone.0166690.s001.tif]

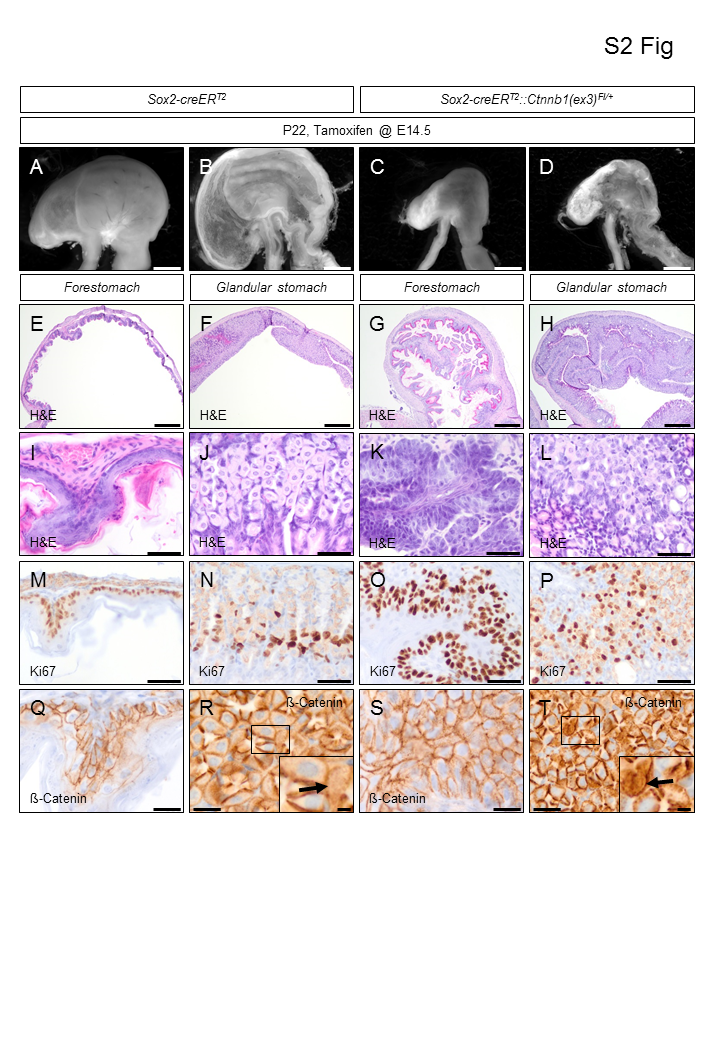

Supplement: S2 Fig — At E14.5 tamoxifen-induced Sox2-creERT2::Ctnnb1(ex3)Fl/+ mice display small stomachs at the time of death compared to controls (whole organ in C vs. A; sliced organ in D vs. B). The stomachs of mutant mice exhibit lumen-narrowing hyperplastic lesions in forestomach (G vs. E) and glandular stomach (H vs. F) parts. The epithelial cells of forestomach lesions show an endophytic growth pattern (K vs. I) with visually enhanced proliferation in Ki67 staining (O vs. M) in comparison to the native forestomach epithelium. The glandular lesions present altered cytoarchitecture and cell morphology compared to the native glandular stomach (L vs. J), whereas ß-Catenin staining intensity seems to be enhanced in these lesions (T vs. R; higher magnification of framed areas in insets). Ki67 staining in glandular lesions and native glands (P vs. N) and ß-Catenin staining in forestomach lesions and native forestomach (S vs. Q) is similar. Scale bars equate to 2 mm in A-D, equate to 500 μm in E-H, equate to 50 μm in I-P, equate to 20 μm in Q-T and equate to 10 μm in insets. (TIF) [file pone.0166690.s002.tif]
